# Reduction of COVID-19 Incidence and Nonpharmacologic Interventions: Analysis Using a US County–Level Policy Data Set

**DOI:** 10.2196/24614

**Published:** 2020-12-21

**Authors:** Senan Ebrahim, Henry Ashworth, Cray Noah, Adesh Kadambi, Asmae Toumi, Jagpreet Chhatwal

**Affiliations:** 1 Harvard Medical School Boston, MA United States; 2 Hikma Health San Jose, CA United States; 3 University of Toronto Toronto, ON Canada; 4 Massachusetts General Hospital Boston, MA United States

**Keywords:** communicable diseases, COVID-19, data set, pandemic, policy, public health, data, intervention, effectiveness, incidence, time series

## Abstract

**Background:**

Worldwide, nonpharmacologic interventions (NPIs) have been the main tool used to mitigate the COVID-19 pandemic. This includes social distancing measures (closing businesses, closing schools, and quarantining symptomatic persons) and contact tracing (tracking and following exposed individuals). While preliminary research across the globe has shown these policies to be effective, there is currently a lack of information on the effectiveness of NPIs in the United States.

**Objective:**

The purpose of this study was to create a granular NPI data set at the county level and then analyze the relationship between NPI policies and changes in reported COVID-19 cases.

**Methods:**

Using a standardized crowdsourcing methodology, we collected time-series data on 7 key NPIs for 1320 US counties.

**Results:**

This open-source data set is the largest and most comprehensive collection of county NPI policy data and meets the need for higher-resolution COVID-19 policy data. Our analysis revealed a wide variation in county-level policies both within and among states (*P*<.001). We identified a correlation between workplace closures and lower growth rates of COVID-19 cases (*P*=.004). We found weak correlations between shelter-in-place enforcement and measures of Democratic local voter proportion (R=0.21) and elected leadership (R=0.22).

**Conclusions:**

This study is the first large-scale NPI analysis at the county level demonstrating a correlation between NPIs and decreased rates of COVID-19. Future work using this data set will explore the relationship between county-level policies and COVID-19 transmission to optimize real-time policy formulation.

## Introduction

In the absence of effective vaccines or therapeutics targeting SARS-CoV-2, nonpharmacologic interventions (NPIs) have been the only effective measures for containing the current COVID-19 pandemic [1-4]. NPIs are grouped into social distancing measures and contact tracing measures [5]. Examples of social distancing measures include closing businesses, closing schools, and quarantining symptomatic persons [6]. Contact tracing involves tracking and following exposed individuals, which requires both testing capabilities and infrastructure to execute [7]. Systematic reviews and modeling studies have demonstrated that each of these NPIs have a variable impact on respiratory virus transmission, depending on how and when they are deployed [1,5,7].

While China was initially slow to implement NPIs, models have shown that China’s social distancing measures were sufficient to control COVID-19 [1,2]. Local governments across China were integral in the successful implementation of NPIs, including diagnostic testing and enforcing social distancing [2]. NPIs have similarly been found to effectively limit COVID-19 across 11 countries in Europe [4], especially Italy [3]. However, the United States has implemented NPIs more variably, which may be related to the higher rates of transmission [8].

In the United States, the first known cases of COVID-19 were reported on January 20, 2020 [9]. However, it was not until March 2020 that individual states responded with NPI policies, and as of April 20, 2020, multiple states still had not implemented stay-at-home orders [10,11]. The limited coordination of national, state, and local responses to COVID-19 led to substantial variation in NPIs at the county level across the United States. As the pandemic continued into late May and early June, states began to roll back measures with incongruent reopening plans. These reopening plans again differed by location and by NPI policy type [9]. As a result, the United States has uniquely experienced wide variations in NPI policy, both geographically and temporally. County-level political party alignments may be relevant to the formulation of local COVID-19 NPI policies; this question has not yet been rigorously assessed. Partisanship in county-level policy has significant ramifications for how policymakers engage stakeholders to realistically implement local policy.

As of November 25, 2020, there are 12,838,102 confirmed COVID-19 cases and 262,847 deaths in the United States [8], both higher totals than those of any other country in the world [8,12]. Preliminary data on social distancing at the state level [13] and limited data on shelter-in-place orders at the county level from bordering communities in Illinois and Iowa [14] have shown that NPIs can be effective, particularly when implemented in a timely manner. However, there remains a need to better understand the effects of NPIs on COVID-19 transmission dynamics at a national scale on the granular county level.

The first objective of this study was to describe the motivation and novel methodology for creating the first large-scale county-level NPI policy data set in the United States. The second objective was to highlight initial findings from this data set to demonstrate its utility for much-needed local NPI analyses. A unique standardized crowdsourcing methodology was deployed to collect time-series data on 7 key NPIs for 1320 US counties. This novel data set was then mined for correlations in combination with publicly available COVID-19 case data, reproduction number (*R_t_*) estimates, and political demographics at the county level. This exploratory analysis illustrates the utility of county-level NPI implementation and analysis in the United States, particularly with the novel data set described herein.

## Methods

### Data Collection

A novel crowdsourcing methodology was implemented by Hikma Health to collect COVID-19 policy data for 1320 US counties from March to July 2020. The data set covers 7 distinct NPI policies, including the most widely deployed and accepted NPIs. For each county, trained volunteers reported a binarized policy status for each NPI policy indicating if/when the following NPI policies were first implemented in each county, along with a timestamp and a reference URL: (1) closure of nonessential workplaces, (2) shelter-in-place/stay-at-home orders, (3) enforcement of shelter-in-place/stay-at-home orders, (4) size restrictions on public gatherings, (5) school closures, (6) public transit closures, and (7) publicly available testing. Table 1 provides a full description of the variables included in the data set. The data set also includes a second timestamp and reference URL for if/when each of the same 1320 US counties terminated the following 2 NPI policies: nonessential workplace closures and shelter-in-place/stay-at-home orders. Given the limits in data collection capacity, these 2 NPI policies were prioritized over the other 5 NPI policies for observation at a second timepoint, as we hypothesized they had a relatively higher likelihood of changing in May-July 2020.

**Table 1:** Variables in the Hikma Health data set of county-level nonpharmacologic intervention policies.

Variable name	Description
fips	County FIPS^a^ geographic code (unique identifier)
testing	Binary coding whether COVID-19 testing is publicly available in the county to any resident without physician referral needed
school	Binary coding whether all schools are closed in the county
shelter	Binary coding whether the county has an active shelter-in-place order, publicly announced by any county official
shelter_enforcement	Binary coding whether the shelter-in-place order is being enforced in the county with fines or other penalties
work	Binary coding whether all “nonessential workplaces” are closed in the county, with any local definition of “nonessential”
event	Binary coding whether public events and gatherings larger than a particular size N are restricted, for any N>1
transport	Binary coding whether any public transportation has been closed down for any public bus, train, shuttle, or ferry routes
X_date	For each policy binary X, the date on which it was first implemented
X_URL	For each policy binary X, the source URL with evidence of the nature and date of the policy
updated	The timestamp for when this data was entered

^a^FIPS: Federal Information Processing Standard.

Because each US county reports its standing COVID-19 NPI policies differently—from county websites to local news outlets and official social media channels—the data collection process cannot be automated and instead requires human review and discernment. From March to July of 2020, 104 volunteers, consisting mostly of health-related graduate degree students and medical professionals, were recruited through COVID-19 project postings, outreach groups, and institutional listservs. Each volunteer was remotely trained to use the same 7-step standard operating procedure to research and collect the aforementioned NPI data on 1320 US counties through standardized online forms, effectively transforming the convoluted county policy landscape into an organized NPI data set with binary yes/no and interval date variables. The free and open-source data set contains corresponding URL references on all counties for quality assurance [15].

In assigning counties to volunteers, we initially prioritized population and then transitioned to COVID-19 incidence as the pandemic unfolded. Specifically, we sourced data for the 500 most populous US counties and then used dynamic 4-day incidence rate calculations to prioritize the remaining counties in real time. For the first 100 counties, every policy and its implementation date was validated manually by double-checking the website URL from which the information was sourced. The same extraction process was repeated for the subsequent 1220 counties, with validation of URLs for an additional randomly selected 10% of completed counties, rendering a volunteer coding accuracy rate over 99%.

### Statistical Analysis

In this study, we conducted time-series correlational analyses combining our county-level NPI data for 1320 US counties with multiple data sources, including daily county COVID-19 cases and deaths sourced from The New York Times. We also assessed correlations with COVID-19 effective *R_t_* estimates from the RT Live project, and political demographics at the county level from the Kaiser Family Foundation and the MIT Election Data and Science Lab.

For optimal visualization and temporally focused analysis on current policies, we constructed a consolidated version of the data set as follows: for each NPI policy in each county, all observations within the last 24 hours were pooled; the mean was calculated for each binary, and binaries above 0.5 were considered positive, while binaries less than or equal to 0.5 were considered negative; the latest date and URL reported were chosen to represent that policy; lastly, a Policy Strength Index (PSI) was calculated as a linear sum of all 7 NPI policy binary variables, with 7 being the maximum and 0 being the minimum possible value.

We then mined this consolidated data set for correlations and distribution differences using standard statistical tests including *T* tests and chi-square tests with a Bonferroni correction applied for multiple hypothesis testing. All analyses were conducted in Python notebooks that are available open source for review and global use under the Apache 2.0 license.

## Results

### Data Set Construction and Access

The full county NPI data set, hereon referred to as the “all policies” data set, yielded 2704 observations of NPI policies described in Table 1 in 1320 counties from all 50 states in the United States. We analyzed the all policies data set (n=2704) as well as the consolidated current version of the data set, hereon referred to as the “current policies” data set (n=1320), containing only the most recent timestamp for county NPIs as described in the *Methods* section. These versions are referred to as the “all_county_ policies” and “county_policies” files, respectively, on GitHub, where they can be freely accessed by the global public, along with reference documentation [15]. The data set is available as both CSV (comma-separated values) and JSON (JavaScript Object Notation) files, indexed by US county FIPS (Federal Information Processing Standard) geographic codes. Each of these files include the binary NPI variables with accompanying interval date variables, corresponding reference URLs, and the timestamps of when the data were collected. The CSV files used for all analyses presented in this paper are included in Multimedia Appendix 1.

### Policy and Case Correlations

**Figure 1:**
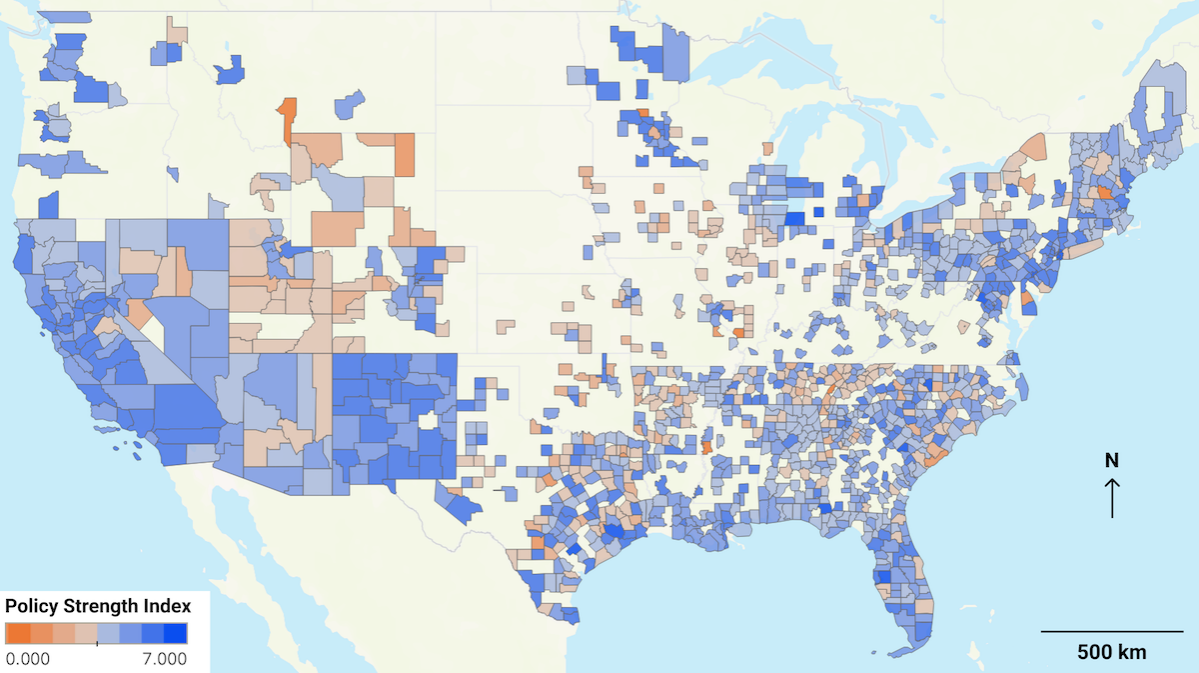
Map showing that county policies are uncorrelated and highly variable between and within states. Policy Strength Index (PSI) for the current policies data set mapped by county indicates that across states, there was wide variability in policy implementation on July 15, 2020. The PSI is calculated as the linear sum of the 7 binary nonpharmacologic intervention policy variables in each county as described in the Methods section.

In the all policies data set, there was a strong positive correlation between nonessential workplace closure and shelter-in-place orders at the county level with a Pearson R of 0.835. All other correlations between individual policies were weak in the all policies data set, with an absolute value of R<0.3. In the current policies data set, the correlation between nonessential workplace closure and shelter-in-place orders weakened to a Pearson R of 0.144, generally in association with the reopening of workplaces without lifting shelter-in-place orders in May-June 2020. In the current policies data set, all other correlations were less than 0.1. Of all policies tested for interstate differences by a one-way ANOVA (analysis of variance), only school closure was not significantly different at a Bonferroni corrected α level of .0071 (*P*=.06); for all other policies *P*<.001. States also exhibited varying degrees of overall intrastate/intercounty variability in PSI, as illustrated in Figure 1.

**Figure 2:**
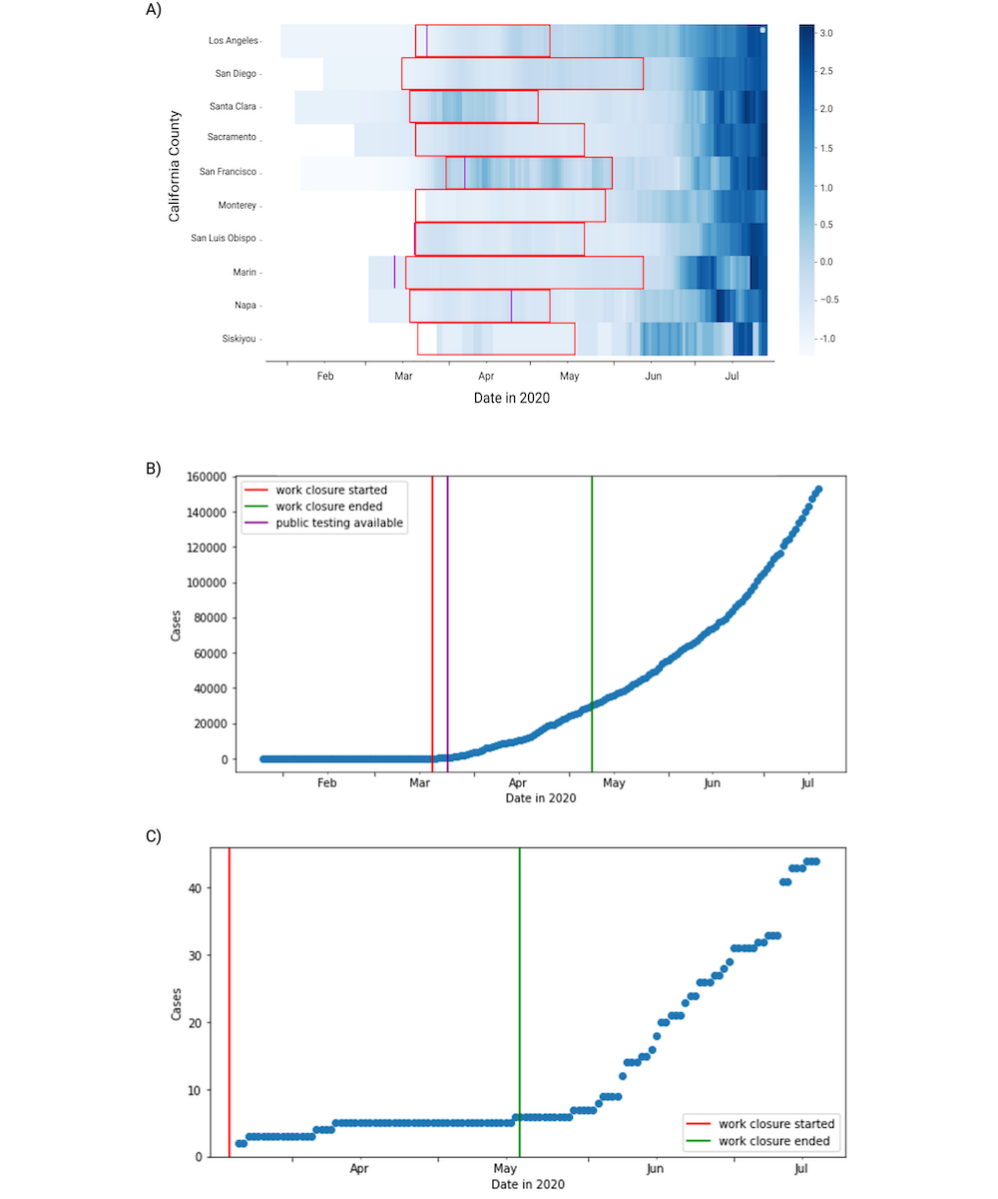
Variability in California county policies in time and in relation to caseloads. (A) Time-series heatmap of the 7-day simple moving average of daily cases plotted for 10 representative California counties in order of descending population, all normalized by county. The red rectangle demarcates the period for which the county had nonessential workplaces closed. The purple line indicates if and when public testing became available. (B) Plot of the total number of county cases as a timeline with county-level nonessential work closure policy and public testing times labeled for Los Angeles County. (C) Plot of the total number of county cases as a timeline with county-level nonessential work closure policy times labeled for Siskiyou County.

We also observed that within states the variability in county NPIs informs the visualization of case growth rates, as shown in Figure 2. For example, in California, the variability in county NPI policies informed the interpretation of timelines showing the 7-day simple moving averages of new cases in each county (Figure 2A). As a general trend, case growth was minimal during the period of workplace closure, highlighted for each county in Figure 2A in the red window, while case growth increased significantly 2-8 weeks after the end of workplace closure. For example, in Los Angeles County, California (shown in Figure 2B), case growth accelerated following the end of the work closure policy; additionally, the start of public testing coincided with the inflection point of case growth. In Siskiyou County, California (shown in Figure 2C), case growth similarly accelerated following the end of workplace closure.

### Policies and Case Growth

**Figure 3:**
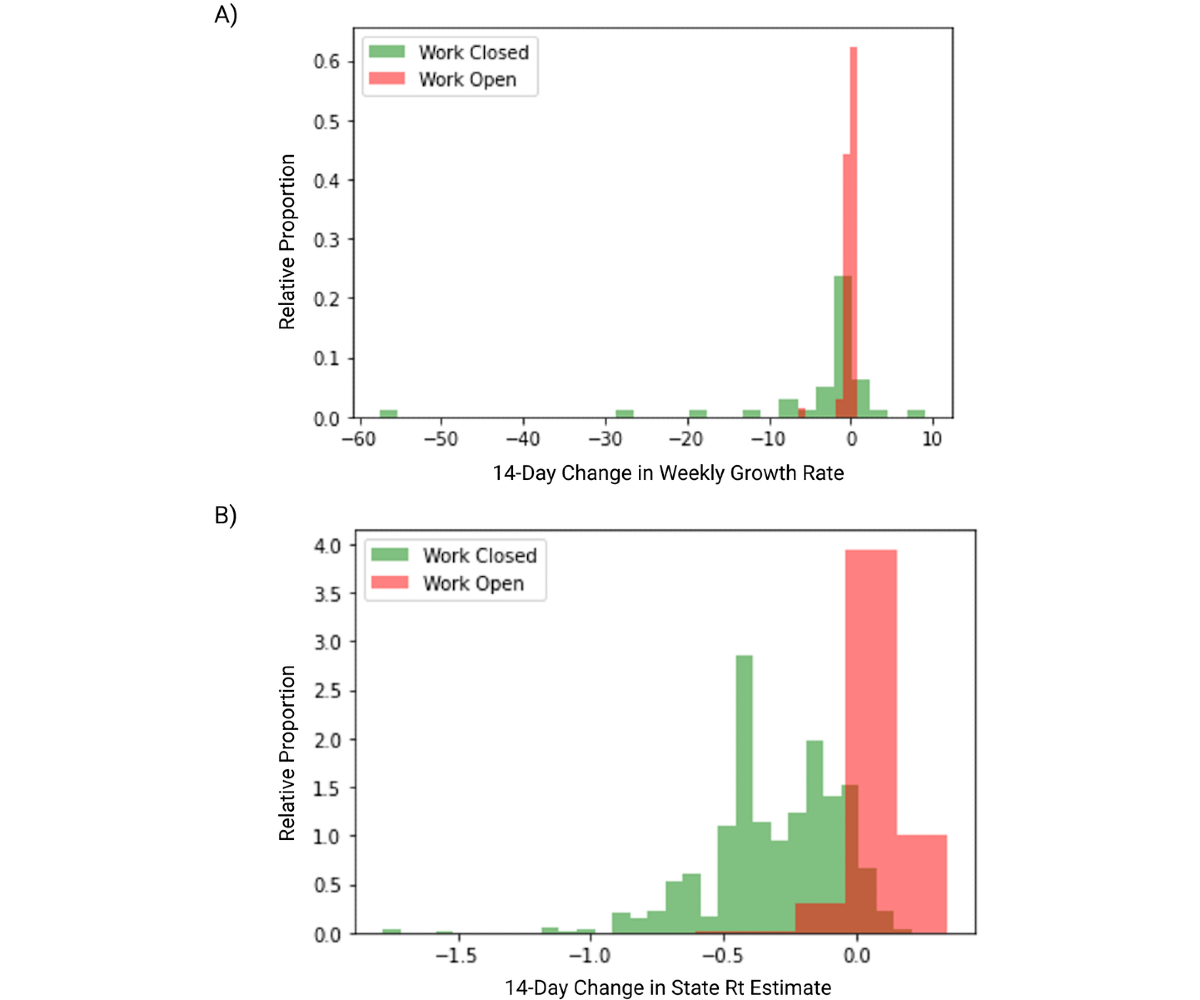
Counties with closed nonessential workplaces and significant declines in growth rate and reproduction number (*R_t_*) following workplace closure. (A) A histogram of the change in weekly case growth rate from the week preceding the date of workplace closure to 14 days later for counties with open versus closed nonessential workplaces. (B) A histogram of the change in RT Live estimates of *R_t_* by state from the date of the workplace policy to 14 days later for counties with open versus closed nonessential workplaces.

As summarized in Figure 3, we assessed whether there are significant differences in the change in weekly case growth in counties where workplaces closed versus those that remained open in the all policies data set. Figure 3A shows a histogram illustrating change in weekly case growth rate from the week preceding the date of workplace closure to 14 days later for counties with open versus closed nonessential workplaces. Weekly case growth rates decreased over the 14 days following a workplace NPI by larger magnitudes for counties that had such nonessential workplace closure policies as compared to those that had openings (*P*=.004; Figure 3A). We also assessed whether there was a significant difference in statewide *R_t_*. This effect of decreasing case propagation following workplace closure was similarly observed in state-level estimates of *R_t_* 14 days after the workplace policy (from RT Live) (*P*<.001; Figure 3B). The effect for *R_t_* was recapitulated at 28 days after the workplace policy (*P*<.001). These effects are statistically significant at our Bonferroni corrected α level of .0071.

The start date of public testing varied in each county both on the absolute date timeline and the timeline relative to other NPIs. To assess whether counties that had free public testing before a workplace policy observation were more likely to have had a workplace closure for that observation, counties were grouped based on their timelines into the categories of no testing, testing after workplace closure, and testing before workplace closure. A chi-square test for significance found no significant difference in workplace openings versus workplace closures for these categories (*P*=.08).

### Policies and Political Alignment Correlations

**Figure 4:**
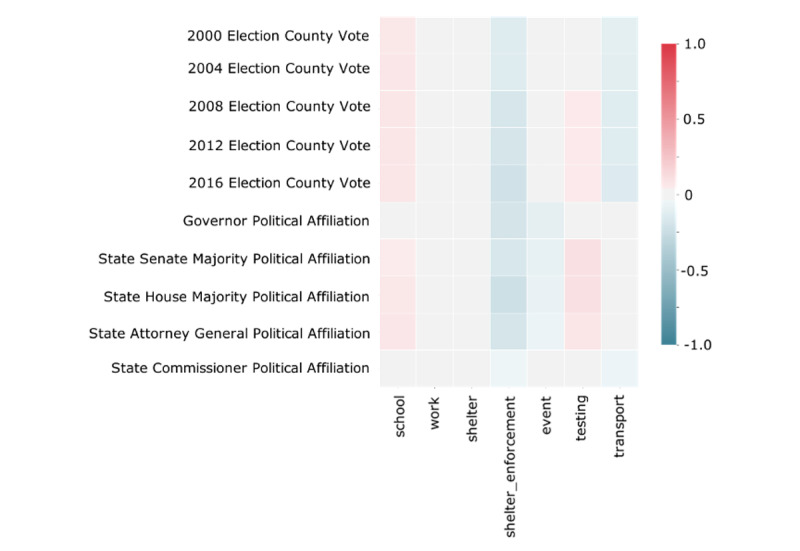
Weak correlation between county policies and county political parties. Correlogram of county-level nonpharmacologic interventions (NPIs) and political election patterns for state and national government for the all policies data set. A positive correlation shown in red indicates a positive correlation between a Republican-oriented election pattern and an NPI.

We observed weak correlations between the political parties of local leadership and electorate and the policy of shelter-in-place enforcement. In the all policies data set, shelter-in-place enforcement was weakly correlated with Democratic party State House leadership (R=0.22) and Democratic voting proportion in the 2016 presidential election (R=0.21); all other correlations had an absolute value of R<0.2 (Figure 4). No correlations greater than 0.2 were observed between county political parties and the dates at which a positive intervention was initially made for a particular NPI policy.

## Discussion

### Principal Results

The most effective measures for countries around the world preventing COVID-19 have been NPIs [1-4]. However, due to policy variations across levels of government, the United States has not demonstrated the same success with NPIs [8]. While some research has been published looking at small collections of neighboring counties, there has not been a comprehensive analysis of NPIs across a wider range of counties or any local level more granular than the state level in the United States [14]. In this study, we identify associations between county level NPI policies and COVID-19 transmission from March 1 to July 20, 2020, using a novel crowdsourced county NPI policy data set.

The first objective of this project was to construct a comprehensive data set that has a broad representation of different NPI policies; has a diverse representation of counties both in geographic distribution and population; and incorporates changes in NPIs over time. Overall, this study rendered a highly granular NPI policy data set with time-series data for 1320 counties from all 50 states in the United States. While there have been comprehensive data sets reviewing NPI policies at the state level [10,11] and some data generated for small clusters of neighboring counties (<100 counties) [14], this novel data set is the most comprehensive publicly available county data set to date. We have openly shared this data set with the aim that it be used by researchers around the United States and the world to further analyze the correlates of NPIs and various COVID-19 epidemiological outcomes. Future research could include a finer-grained analysis and modeling of the relationships between COVID-19 cases, deaths, and NPI policies, as well as the explorations of the relationship between NPIs and outcomes including economic status, health care utilization, and social inequities.

The second objective of this project was to analyze this data set and report preliminary findings. We found that across the United States, there was significant variability in NPI policy implementation among counties, both within and among states. NPI policies themselves for any given county are largely uncorrelated, with the singular exception of workplace closure and shelter-in-place NPIs. This finding supports previous research that has also found wide variation in policies across states, which was associated with statistically significant differences in rates of COVID-19 transmission [13]. While no causal link has been demonstrated, these strong correlations are further supported by international studies that have compared similarly sized territories within countries [2,3].

We also found that there was significant variation in NPI policies across counties within states. We displayed 10 representative California counties as an example, which show the relationship between the rate of COVID-19 cases and timing of when individual California counties closed workplaces, reopened workplaces, and started public testing. These results show that the implementation of NPI policies varied when they were enacted in response to COVID-19 case rates across counties. As a consistent trend, we observed that for both heavily populated urban counties like Los Angeles County (10 million residents) and sparsely populated rural counties like Siskiyou County (44,000 residents), the end of a workplace closure appears to precede an increase in cases by 2-6 weeks. This heatmap timeline could be confounded by when public testing started in each county and the rate at which counties were able to test. While we have recorded the date at which public testing became available in each county, as displayed in Figure 2, the rate of testing by county remains generally unknown.

The final and most notable finding of this analysis is that there are statistically significant positive correlations between county workplace closures and decreases in subsequent COVID-19 case growth, as shown in Figure 3. We found that across all counties, those with work closures had significantly lower rates of subsequent COVID-19 case growth compared to counties that did not. We emphasize that these results do not in any way demonstrate causation but rather a temporally informative correlation between NPI policy and COVID-19 rates. Nevertheless, these findings align with others highlighting the relationship between increased NPI policies and subsequently declining growth rates of COVID-19 at the county level [14]. Our Hikma Health data set also has the potential to be used in further temporal modeling, particularly to predict *R_t_* for counties across the United States. Our data set particularly lends itself to a clustering analysis to assess the relationship between NPI policies and cases in demographically similar counties, which will be helpful to local policymakers.

Current research on political affiliation and attitudes toward COVID-19 policies has shown that differences between Democrats and Republicans are more significant than differences across race or gender [16]. Reports from the Pew Research Center have shown that Democrats are more likely to see COVID-19 as a serious threat [17]. In order to understand the political landscape in which these policies are being implemented, we analyzed both the political parties in charge of counties and the political electorate in each county. As shown in Figure 4, we found a relatively weak correlation between Democratic county governments and electorates and shelter-in-place policy enforcement (no significant correlations). However, our finding of relatively limited differences by political party is consistent with a generally high public support across the political spectrum for NPI policies to reduce COVID-19 spread [18]. These preliminary results should be further analyzed to understand the relationship between political party and NPI policy choices.

### Limitations

There are a number of important limitations to note about our study. Firstly, our data collection, while rigorous, is affected by a number of factors both inherent to the study and external that could skew outcomes. Even though data collectors were well trained and used standardized methods, the estimated date of policy changes could be highly variable, particularly for counties with limited or conflicting information available online. Thus, an important next step will be to fully validate this Hikma Health data set by double coding all 1320 counties and subsequent reconciliation of discordant datapoints. In addition, external and contemporaneous factors such as rates of testing and the degree to which the public actually adhere to NPI guidelines are not addressed by our binarized data set. In subsequent analyses to address these factors, we aim to integrate testing rates and mobility data as these data sets become available.

Secondly, the Hikma Health data set comprises 2 timepoints for 2 of the 7 NPI policies originally assessed. The analyses presented in Figures 2 and 3 are subject to the caveat that changes in the other 5 unrecorded NPI policies may theoretically confound any associations. Of these NPI policies, enforcement of shelter-in-place/stay-at-home orders, school closures, and publicly available testing were unlikely to change before August 2020, whereas size restrictions on public gatherings and public transit closures may have been reversed and therefore might be more significant confounds.

Finally, our analysis identifies correlations in the data set without any implication of causality. In order to establish causation, NPI policies would have to be implemented as a coordinated randomized controlled trial across counties, which is unlikely. In lieu of such a study, our group and others will build temporal predictive models using this data set to test the potential effects of NPI policies.

Despite these limitations, our study is the first to identify correlations between county-level NPI policies and subsequent COVID-19 growth rates across the United States, including over 1000 counties from all 50 states. Our novel data set enabled us to consistently describe correlations for counties across the United States, compared to previous studies conducted on a more limited and thus less representative scale [13,14]. Our data collection methodology allowed for the evaluation and validation of data across geography and time. As the pandemic continues, future research should continue to investigate the relationship between NPI policies, COVID-19 case rates, and the factors that may influence implementation such as political affiliation, culture, and social structures.

### Conclusion

As COVID-19 cases continue to climb across the United States, we anticipate that local leadership at the state and county levels will need to devise informed and relevant policies to limit local spread. Our findings suggest that there is substantial variation in NPI implementation and termination at the county level, both between and within states, reflecting an inconsistent policy approach. We also found positive correlations between implementing a workplace closure NPI and lower future rates of COVID-19, supporting previous national and international studies suggesting that NPI policies like workplace closure reduce COVID-19 transmission [1-4,13,14]. Taken together, this growing body of literature suggests that NPI policies at multiple levels, and especially at the local level of the county, play a role in limiting the effects of the COVID-19 pandemic.

## Appendix

Multimedia Appendix 1 HikmaHealthCountyData.zip contains two CSV data files analyzed in this paper: 'all_county_policies.csv' with all policies reported for each county, and 'county_policies.csv' with a synthesized policy summary for each county (generated by process described in the manuscript).

## Abbreviations

ANOVA: analysis of variance
CSV: comma-separated values
FIPS: Federal Information Processing Standard
JSON: JavaScript Object Notation
NPI: nonpharmaceutical intervention
PSI: Policy Strength Index
*R_t_*: reproduction number
